# Rust expression browser: an open source database for simultaneous analysis of host and pathogen gene expression profiles with expVIP

**DOI:** 10.1186/s12864-021-07488-3

**Published:** 2021-03-09

**Authors:** Thomas M. Adams, Tjelvar S. G. Olsson, Ricardo H. Ramírez-González, Ruth Bryant, Rosie Bryson, Pablo Eduardo Campos, Paul Fenwick, David Feuerhelm, Charlotte Hayes, Tina Henriksson, Amelia Hubbard, Radivoje Jevtić, Christopher Judge, Matthew Kerton, Jacob Lage, Clare M. Lewis, Christine Lilly, Udi Meidan, Dario Novoselović, Colin Patrick, Ruth Wanyera, Diane G. O. Saunders

**Affiliations:** 1 John Innes Centre, Norwich Research Park, Norwich, NR4 7UH UK; 2 RAGT Seeds Ltd, Grange Road, Ickleton, Essex, CB10 1TA UK; 3 BASF SE, Agricultural Centre, Limburgerhof, Germany; 4 INTA EEA Bordenave, Bordenave, 8187 Buenos Aires, Argentina; 5 Limagrain UK Ltd, Woolpit, IP30 9UP UK; 6 Syngenta Seeds Ltd, Hill Farm Road, Cambridgeshire, CB22 4QT UK; 7 Elsoms Wheat Ltd, Spalding, Lincolnshire, PE11 1QG UK; 8 Lantmännen Lantbruk, Svalöv, Sweden; 9 NIAB, Cambridge, CB3 0LE UK; 10 Institute of Field and Vegetable Crops, Novi Sad, Serbia; 11 DSV United Kingdom Ltd, Banbury, Oxfordshire, OX17 1FE UK; 12 KWS UK Limited, Hertfordshire, SG8 7RE UK; 13 Frontier Agriculture, Witham St Hughs, Lincolnshire, LN6 9TN UK; 14 Hazera Seeds Ltd., Berurim M.P Shikmim, 7983700 Tel Aviv-Yafo, Israel; 15 Agricultural Institute Osijek, Osijek, Croatia; 16 Masstock Arable (UK) Ltd. (trading as Agrii), Andoversford, Gloucestershire, GL54 4LZ UK; 17 Kenya Agricultural and Livestock Research Organization, Njoro, Nakuru, Kenya

**Keywords:** RNA-Seq, expVIP, Gene expression browser, Wheat yellow rust, *Puccinia striiformis* f. sp. *tritici*, Transcriptomics, Open science

## Abstract

**Background:**

Transcriptomics is being increasingly applied to generate new insight into the interactions between plants and their pathogens. For the wheat yellow (stripe) rust pathogen (*Puccinia striiformis* f. sp. *tritici*, *Pst*) RNA-based sequencing (RNA-Seq) has proved particularly valuable, overcoming the barriers associated with its obligate biotrophic nature. This includes the application of RNA-Seq approaches to study *Pst* and wheat gene expression dynamics over time and the *Pst* population composition through the use of a novel RNA-Seq based surveillance approach called “field pathogenomics”. As a dual RNA-Seq approach, the field pathogenomics technique also provides gene expression data from the host, giving new insight into host responses. However, this has created a wealth of data for interrogation.

**Results:**

Here, we used the field pathogenomics approach to generate 538 new RNA-Seq datasets from *Pst*-infected field wheat samples, doubling the amount of transcriptomics data available for this important pathosystem. We then analysed these datasets alongside 66 RNA-Seq datasets from four *Pst* infection time-courses and 420 *Pst*-infected plant field and laboratory samples that were publicly available. A database of gene expression values for *Pst* and wheat was generated for each of these 1024 RNA-Seq datasets and incorporated into the development of the rust expression browser (http://www.rust-expression.com). This enables for the first time simultaneous ‘point-and-click’ access to gene expression profiles for *Pst* and its wheat host and represents the largest database of processed RNA-Seq datasets available for any of the three *Puccinia* wheat rust pathogens. We also demonstrated the utility of the browser through investigation of expression of putative *Pst* virulence genes over time and examined the host plants response to *Pst* infection.

**Conclusions:**

The rust expression browser offers immense value to the wider community, facilitating data sharing and transparency and the underlying database can be continually expanded as more datasets become publicly available.

**Supplementary Information:**

The online version contains supplementary material available at 10.1186/s12864-021-07488-3.

## Background

Transcriptomic studies that map fluctuations in the full complement of RNA transcripts, have revolutionized genome-wide gene expression analysis. For plant pathogens, the simultaneous analysis of host and pathogen transcriptomes has enabled many long-standing questions in plant pathology to be addressed particularly regarding how both organisms modulate gene expression at the host-pathogen interface [1]. This has provided new insight into the changes in gene expression profiles of both host and pathogen species. For instance, examination of the rice blast fungus *Magnaporthe oryaze* infecting rice plants identified a set of differentially expressed genes in both the host and the pathogen with more drastic expression changes in incompatible than compatible interactions [2]. Additionally, such analyses have revealed the importance of gene expression polymorphisms. For instance, the gain of virulence for the *Phytophthora infestans* EC-1 lineage on potato carrying *Rpi-vnt1.1* was shown to be due to lack of expression of the corresponding effector *Avrvnt1* [3]. Hence, RNA-based sequencing (RNA-Seq) is being increasingly applied to study the plant-microbe interface, providing an unbiased quantification of expression levels of transcripts that is relatively inexpensive, highly sensitive, and provides high-throughput, high resolution data.

For the wheat yellow (stripe) rust pathogen (*Puccinia striiformis* f. sp. *tritici*, *Pst*) the application of RNA-Seq approaches has proved particularly valuable, overcoming the barriers associated with its obligate biotrophic nature. For instance, evaluating gene expression in wheat plants infected by *Pst* and the powdery mildew pathogen *Blumeria graminis* f. sp. *tritici* (*Bgt*), identified commonalities and differences in the metabolic pathways that were differentially expressed in response to infection through an EST-based approach [4]. Another study, evaluating host responses throughout a time-course of *Pst* infection identified temporally coordinated waves of expression of immune response regulators in wheat that varied in susceptible and resistant interactions [5]. Furthermore, as a pathogen of global concern, an RNA-Seq based surveillance approach was developed for *Pst* called “field pathogenomics” that has been used to study its population dynamics at an unprecedented resolution [6]. The application of this methodology in the UK uncovered recent changes in the population composition of *Pst*, whilst also revealing varietal and temporal associations of specific *Pst* races (pathotypes) that can help inform disease management [6, 7]. As a dual RNA-Seq approach applied directly to *Pst* infected leaf samples it also provides gene expression data from the host side of the interaction giving new insight into host responses [8]. These approaches generate a wealth of RNA-Seq data that is exceptionally valuable but difficult for those without specialist skills to access, which also inhibits reproducibility of transcriptomic studies.

Currently, the standard for open sharing of RNA-Seq data is to ensure raw reads are deposited in public repositories such as NCBIs Sequence Read Archive (SRA) [9]. However, utilising this data requires specialist bioinformatic expertise and often the use of high-performance computing systems. To overcome this, a series of gene expression browsers have been developed to enable interactive exploration of expression data [10–12]. However, the amount of data included within these databases for *Pst* is limited. The recently released fungi.guru transcriptomic database contains data for *Pst* gene expression from a limited number of samples, however it does not include the large number of field samples currently available or expression profiles for the wheat host [13]. Evaluation of gene expression levels in the wheat host can be undertaken separately using the wheat expression browser; an interactive gene expression browser that uses the RNA-Seq data analysis and visualisation platform expVIP (expression Visualisation and Integration Platform) [14]. However, although this browser hosts a number of RNA-Seq datasets from *Pst*-infected wheat tissue, this data has only been aligned to the wheat host transcriptome, inhibiting the exploration of gene expression profiles on the pathogen side of the interaction. For wheat, the expVIP browser has been extremely useful in providing an open access interface for the visualisation of RNA-Seq datasets. This has been instrumental in improving the understanding of the role of a variety of different wheat genes, such as the iron transporter *TaVIT2* and its potential role in biofortification [15] and the role of *TEOSINTE BRANCHED1* in the regulation of inflorescence architecture and development [16]. As the underlying software is also publicly available [17], an instance was recently developed to support analysis of fruit development for a wild blackberry species (*Rubus genevieri*) and cultivated red raspberry (*Rubus idaeus* cv. *prestige*) [18]. However, it has yet to be specifically applied to support analysis of plant-microbe interactions.

Here we present the first instance of a gene expression browser using the expVIP software that enables simultaneous exploration of both host and pathogen gene expression profiles. Focused on *Pst*, in this initial release we collated and processed 958 RNA-Seq datasets from use of the field pathogenomics methodology and 66 RNA-Seq datasets from *Pst* infection time course experiments for incorporation into the rust expression browser. With 538 of these RNA-Seq datasets generated herein, this has doubled the amount of RNA-Seq data available for this pathosystem and represents the largest collection of processed RNA-Seq datasets available for any of the three wheat rust pathogens. Using our new browser, the underlying database of gene expression values can be easily accessed for both *Pst* and its wheat host under an array of experimental conditions and across developmental stages. We show the utility of the browser for the analysis of putative virulence genes from the pathogen and the response of the host plant to *Pst* infection. This illustrates the immense value of analysing a broad set of RNA-Seq data to provide insight into gene expression regulation during host-pathogen interactions.

## Construction and content

### Generating RNA-Seq data and its incorporation into the rust expression browser

To generate data for incorporation into the *Pst* expression browser we first used a set of 538 *Pst*-infected plant samples that were collected across 30 countries from 2014 to 2018 (Supplementary Table S1). *Pst*-infected wheat leaf samples were collected and initially stored in RNAlater™ solution to preserve nucleic acid integrity (Thermo Fisher Scientific, United Kingdom) as previously described [6]. Total RNA was extracted from each sample, quality checked using an Agilent 2100 Bioanalyzer (Agilent Technologies, United Kingdom) and sequencing libraries prepared using an Illumina TruSeq RNA Sample Preparation Kit (Illumina, United Kingdom). Samples were subjected to RNA-Seq analysis using Illumina short read sequencing either at the Earlham Institute (United Kingdom; until April 2017) or Genewiz (USA; since April 2017) using the Illumina HiSeq 2500.

**Fig. 1:**
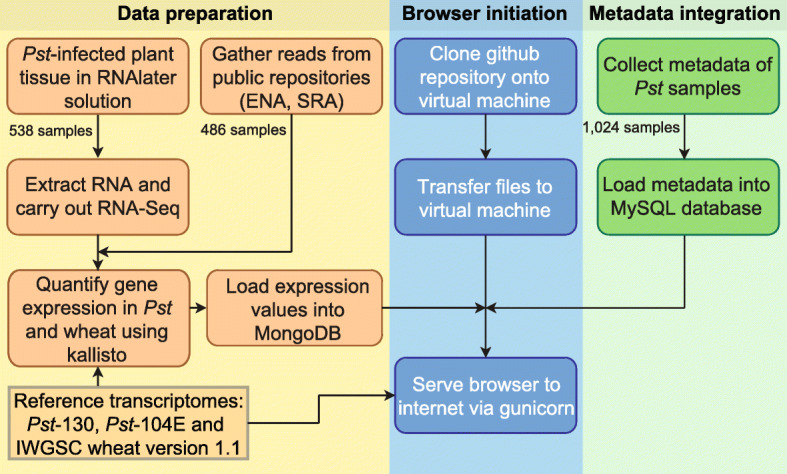
Flowchart illustrating the construction of the rust expression browser. RNA-Seq data was collated from 1024 *Pst* samples and pseduoaligned to the *Pst* reference transcriptomes (isolates *Pst*-130 [19] and *Pst*-104E [21]) and wheat transcriptome version 1.1 [25] using kallisto [26], generating gene expression values (“Data preparation”). Metadata was gathered for each sample and loaded into a MySQL database. Data included where available (i) host species and variety, (ii) host developmental stage, (iii) host tissue type, (iv) fungicide treatment, (v) level of infection, and (vi) collection date and location information (“Metadata integration”). The publicly available expVIP code was cloned from GitHub and transferred to a virtual machine. Metadata, gene expression values and the reference transcriptome were then integrated into the rust expression browser, served to the internet using gunicorn (“Browser initiation”). All computer code used is available as a github repository [27, 28] and metadata files are available via figshare [29]

To further expand this initial dataset, we also identified a total of 486 RNA-Seq datasets from four previously published *Pst* infection time-courses (66 datasets) and *Pst*-infected plant field samples (420 datasets) [5–7, 19–24]. Each of the 1024 transcriptomic datasets were independently pseudoaligned to two *Pst* reference transcriptomes: *Pst* isolate *Pst*-130 [19] and isolate *Pst*-104E [21]. As the vast majority of samples (1004) were from *Pst*-infected wheat tissue, these datasets included both wheat and pathogen-derived reads, thereby samples were also pseudoaligned to version 1.1 of the wheat transcriptome [25]. To facilitate the processing of large numbers of RNA-Seq datasets, the kallisto aligner version 0.42.3 is used in the expVIP framework as an ultra-fast algorithm that was specifically developed for processing large-scale RNA-Seq datasets of short reads for gene expression quantification [26]. Transcript abundances were determined from the kallisto pseudoalignments and incorporated into a MongoDB database for integration into the rust expression browser (Fig. 1).

### Construction of the rust expression browser

The rust expression browser makes use of a modified version of the expVIP code previously used for the wheat expression browser [14] available as a github repository [30]. This repository was cloned onto a virtual machine running CentOS 7, kernel version 3.10.0–1062.12.1.el7.x86_64. Metadata information for the samples was loaded into a MySQL database client version 5.5.68-MariaDB and expression values generated using kallisto [26] were loaded into a MongoDB database version 4.0.22 (Fig. 1). Transcript abundances, alongside the metadata and reference transcriptomes, was then integrated into the expVIP database instance for *Pst* [31]. This instance was then made accessible to web browsers through the use of gunicorn v5.5.3.

## Utility and discussion

### The rust expression browser allows exploration of a broad array of *Pst*-based RNA-Seq datasets

**Fig. 2:**
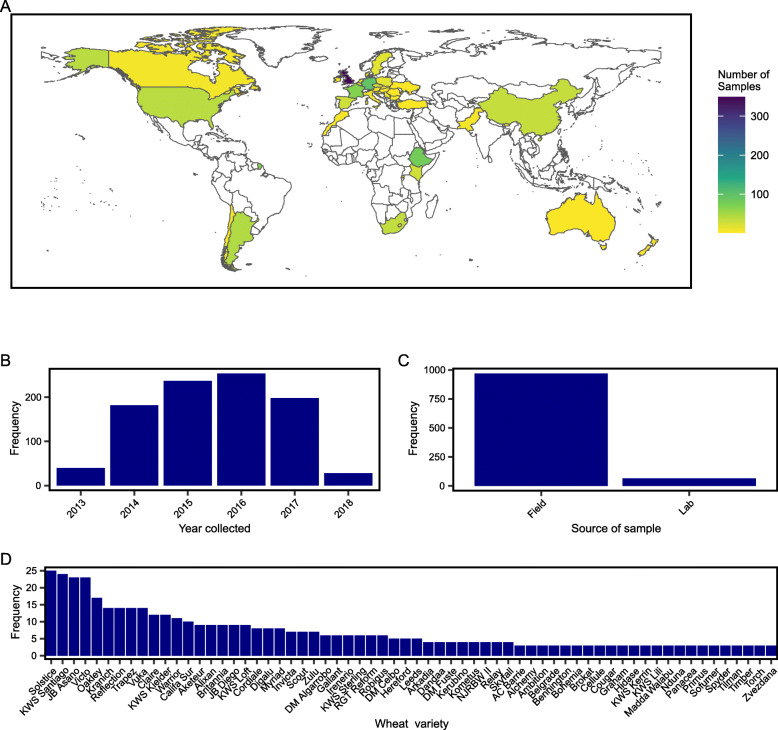
*Pst* RNA-Seq samples were obtained from diverse geographic locations, experimental conditions and wheat varieties. **a** RNA-Seq datasets were generated from *Pst*-infected plant samples collected from all wheat growing continents, with a large number (642 samples) from Europe and especially the UK (334 samples). The map was created in R version 4.0.2 [35], using packages rnaturalearth version 0.1.0 [36], rnaturalearthdata version 0.1.0 [37] and rgeos version 0.5–5 [38]. **b** The 939 *Pst* RNA-Seq datasets from field collected *Pst*-infected plant samples were collected between 2013 and 2018. **c** The vast majority (92%) of *Pst* RNA-Seq datasets were generated from field collected *Pst*-infected plant samples. **d** *Pst*-infected field plant samples were collected from 64 wheat varieties where the variety could be confirmed. Those wheat varieties with at least 3 samples are illustrated. Varieties were confirmed based on their presence in the EU crop variety database [33] or the CIMMYT pedigree database [34]

The inclusion of detailed metadata alongside each *Pst* RNA-Seq dataset within the expVIP framework enables users to easily group data and filter based on categories of interest (Fig. 1; Supplementary Figure S1). To maximise the value of the interface, metadata was gathered for each sample that included where available (i) host species and variety, (ii) host developmental stage, (iii) host tissue type, (iv) fungicide treatment, (v) level of infection, and (vi) collection date and location information. Among the 1024 transcriptomic datasets, 939 represented *Pst*-infected field samples that were collected across all wheat growing continents between 2013 and 2018, with a large number (642 samples) from Europe and especially the UK (334 samples; Fig. 2a). Over 92% of the 939 *Pst*-infected field samples were collected between 2014 and 2017 (Fig. 2b-c), which follows a period of change in the *Pst* population dynamics in Europe and hence a flurry of *Pst* surveillance activities and sample collection [32]. For samples where the wheat variety was recorded, this was cross referenced with the EU plant variety database [33] and CIMMYT variety pedigree database [34]. If a variety could be confirmed in either database, it was also included in the browser metadata (Fig. 2d).

### Simultaneous analysis of multiple RNA-Seq experiments can provide new insight into the expression dynamics of *Pst* virulence factors

**Fig. 3:**
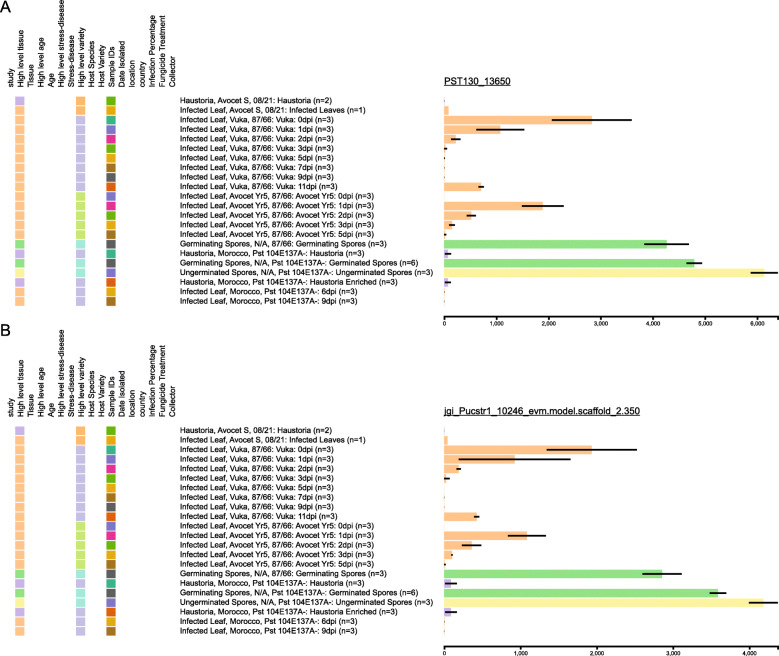
A predicted virulence enhancing *Pst* CAZY gene is expressed early in the infection process. Gene expression analysis across several time courses of *Pst* infection confirmed the expression of a gene encoding a putative carbohydrate-active enzyme (CAZY) termed *Pst_13661* early during the infection process [40] and suggested a second peak of expression at 11 days post-inoculation (dpi). Analysis was undertaken following identification of the corresponding gene in the two *Pst* reference transcriptomes: *Pst*-130 (a) and *Pst*-104E (**b**)

To explore the utility of the rust expression browser, we examined several genes of interest within the browser interface. For *Pst*, we focused on evaluating the expression of a gene (*Pst_13661*) that was recently reported to encode a putative carbohydrate-active enzyme (CAZY) that are known to be conserved across biotrophic fungi [39]. It was reported that *Pst_13661* is able to suppress chitin-induced cell death and, through RT-qPCR analysis, to be highly induced early in infection progression, particularly at 12- and 48-h post inoculation (hpi), with a reduction at 72 and 96 hpi [40]. To evaluate *Pst_13661* expression across all four time-courses of *Pst* infection within the rust expression browser [5, 19–21], we first identified the corresponding gene from the two *Pst* reference genomes using BLASTn [41, 42] conducted via implementation of SequenceServer version 1.0.12 [43] on the main page of the browser (PST130_13650 and jgi_Pucstr1_10246_evm.model.scaffold_2.350; Fig. 3). In accordance with the RT-qPCR analysis, high levels of expression were detected in all cases early in the infection process that was abolished 3 days post-inoculation (dpi). However, within the expression browser we were also able to investigate expression in specific *Pst* developmental stages and across the full infection process in multiple independent experiments. This analysis showed that the gene was highly expressed in ungerminated and germinated urediniospores, had low levels of expression in isolated haustoria, and increased in expression at 11 days post inoculation (dpi) to a level similar to that observed between 1 and 2 dpi. This may suggest a function for this gene later in the infection process or reflect its high level of expression in urediniospores that would begin formation by 11 dpi. The ability to rapidly assess gene expression across an array of time-points, *Pst* developmental stages and experiments provides new insight into the expression of *Pst_13661* without the need for further lengthy and labour-intensive RT-qPCR analysis.

### Gene expression analysis of wheat responses to *Pst* infection

**Fig. 4:**
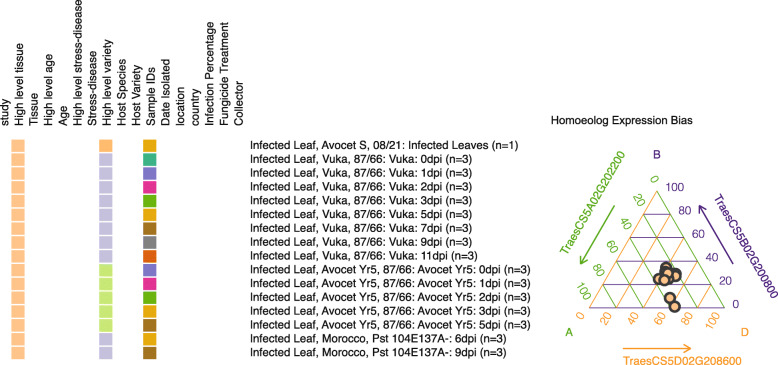
*TaEDS1* expression is biased towards the D genome copy during *Pst* infection. *TaEDS1* expression was analysed in *Pst*-infected leaf samples from time course experiments, illustrating an expression bias towards the D genome copy (46.64% ± 0.01), with the lowest level of expression in the B genome copy (25.05% ± 0.02)

As the vast majority of *Pst* RNA-Seq datasets incorporated in the browser were generated from *Pst*-infected wheat tissue, gene expression analysis can also be undertaken on the wheat host during *Pst* infection. To illustrate this, we examined the Enhanced Disease Susceptibility 1 (*EDS1*) gene homologues in wheat. *EDS1* was first defined in *Arabidopsis thaliana* and is essential for *R*-gene mediated and basal defence responses to biotrophic pathogens such as *Hyaloperonospora arabidopsidis* (formerly *Peronospora parasitica*) [44, 45]. Recently, the homologous genes in wheat have been identified as being important in the response of wheat to infection with the powdery mildew pathogen *Bgt* [46]. As a polyploid, bread wheat (*Triticum aestivum*) typically contains three copies of most genes with one each on the A, B and D chromosomes. It has been shown that the expVIP pipeline is able to accurately distinguish the expression of the three homeologues [14]. Hence, using the expVIP-derived rust expression browser we analysed the expression of the three homeologues of *EDS1* in wheat during *Pst* infection across the samples from four infection time-courses that contained wheat tissue. This analysis revealed that overall expression of the wheat homeologues of *EDS1* tended to be biased towards the D genome copy (46.64% ± 0.01) with the expression of the B genome copy at the lowest level (25.05% ± 0.02; Fig. 4). This is in contrast to that reported for *Bgt*-infected wheat plants, where the highest level of expression was observed in the B genome copy and lowest in the D genome copy. This observation could lead to a greater understanding of the response of wheat to biotrophic pathogens through further analysis of the response of the *EDS1* genes to different pathogen species.

**Fig. 5:**
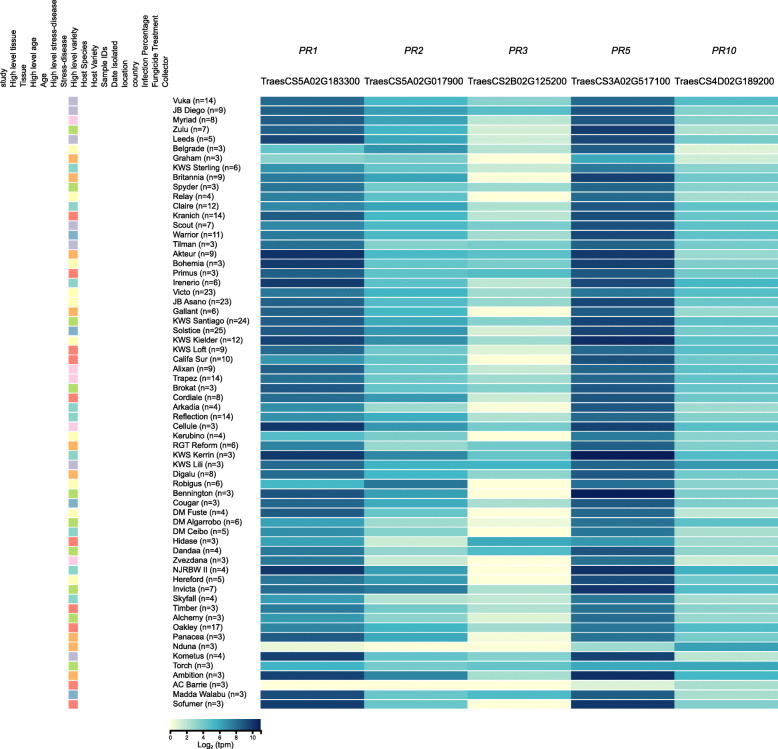
The pathogenicity related (*PR*) genes *PR1* and *PR5* were highly expressed during *Pst* infection. A subset of *Pst*-infected wheat field and laboratory samples was examined for expression of *PR1* (TraesCS5A02G183300), *PR2* (TraesCS5A02G017900), *PR3* (TraesCS2B02G125200), *PR5* (TraesCS3A02G517100) and *PR10* (TraesCS4D02G189200). Gene expression is presented as a heatmap and includes only those samples where the wheat variety could be confirmed and at least three entries were present in the browser

We also evaluated the expression of pathogenesis related (*PR*) genes across 939 *Pst*-infected field and 19 *Pst*-infected laboratory wheat samples. In wheat, *PR* gene expression has been reported to be cultivar and pathogen specific, with different *PR* gene expression patterns also associated with resistance to different *Puccinia* species [47]. We examined the expression of *PR1* (TraesCS5A02G183300), *PR2* (TraesCS5A02G017900), *PR3* (TraesCS2B02G125200), *PR5* (TraesCS3A02G517100) and *PR10* (TraesCS4D02G189200) across all *Pst*-infected field samples where the variety had been confirmed and at least 3 entries were present in the browser (Fig. 5). *PR1* and *PR5* were the most highly expressed across all samples, whilst *PR3* showed the lowest expression level. However, we also found a large amount of variation in the expression of each gene across different varieties, potentially reflecting a difference in their response to *Pst* infection.

## Conclusions

Here we report the development of a novel database and tool for simultaneous ‘point and click’ access to gene expression profiles for *Pst* and its wheat host during the infection process. With 1024 *Pst* samples from an array of developmental stages, experimental conditions and wheat varieties, this browser provides rapid access to gene expression values that can be used as an alternative to lengthy RT-qPCR assays where appropriate. With the largest database of processed RNA-Seq datasets available for any of the three wheat rust pathogens, the rust expression browser offers immense value to the wider community. We have shown how this browser can be used to provide new insight into the expression profiles for *Pst* virulence genes over time and the host plants response to *Pst* infection. As new RNA-Seq data becomes available this can easily be incorporated into the browser, continuing to enhance studies into the *Pst*-wheat interaction.

## Supplementary Information

**Additional file 1: Supplementary Table S1.** Metadata for the *Pst* samples used in this study.

**Additional file 2: Supplementary Figure S1.** Flexible filtering can be applied in the wheat expression browser. All expression values presented are for the candidate CAZY effector *Pst_13661* in the *Pst*-130 genome (termed PST130_13650) [40]. Illustration of data filtered to display only samples from a single study [22] (**A**) or data only from germinated and ungerminated urediniospores and purified haustoria (**B**).

## Data Availability

All raw sequence data used in this study are available at the European Nucleotide Archive (ENA): PRJEB39201, PRJEB33109, PRJEB31334, PRJEB15280 and PRJEB12497; and the NCBI SRA: PRJNA256347, PRJNA181960, PRJNA176472 and PRJNA396589 (Supplementary Table S1). All computer code used in this analysis are available as github repositories [27, 28] and metadata files are available via figshare [29]. The rust expression browser is available at: http://www.rust-expression.com. JavaScript should be enabled; we recommend use of Chrome, Firefox, Safari and Edge web browsers for optimal performance.

## Abbreviations

*Pst*: *Puccinia striiformis* f. sp. *tritici*
RNA-Seq: RNA-based sequencing
*Bgt*: *Blumeria graminis* f. sp. *tritici*
EST: Expressed sequence tag
NCBI: National Center for Biotechnology Information
SRA: NCBIs Sequence Read Archive
expVIP: expression Visualisation and Integration Platform
CIMMYT: International Maize and Wheat Improvement Center
EU: European Union
CAZY: Carbohydrate-active enzyme
RT-qPCR: Quantitative reverse transcription PCR
BLAST: Basic Local Alignment Search Tool
*EDS1*: Enhanced Disease Susceptibility 1 gene
*PR*: Pathogenesis related genes
