# Supplementary material for: Rust expression browser: an open source database for simultaneous analysis of host and pathogen gene expression profiles with expVIP
Source: BMC Genomics. 2021 Mar 9;22:166. doi: 10.1186/s12864-021-07488-3 (PMC7941908; doi:10.1186/s12864-021-07488-3)
Supplement: Supplementary file 2 — Additional file 2: Supplementary Figure S1. Flexible filtering can be applied in the wheat expression browser. All expression values presented are for the candidate CAZY effector Pst_13661 in the Pst-130 genome (termed PST130_13650) [40]. Illustration of data filtered to display only samples from a single study [22] (A) or data only from germinated and ungerminated urediniospores and purified haustoria (B). [file 12864_2021_7488_MOESM2_ESM.pdf]

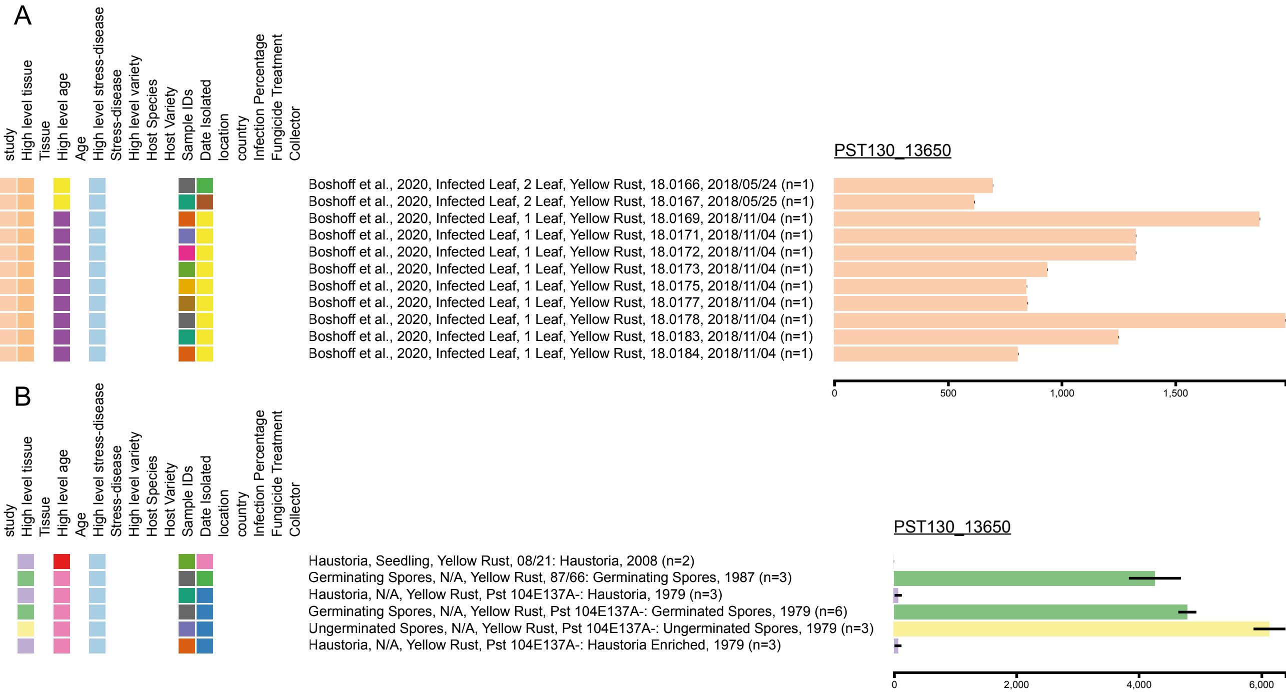
A
study
High level tissue
Tissue
High level age
Age
High level stress-disease
Stress-disease
High level variety
Host Species
Host Variety
Sample IDs
Date Isolated
location
country
Infection Percentage
Fungicide Treatment
Collector
PST130_13650
Boshoff et al., 2020, Infected Leaf, 2 Leaf, Yellow Rust, 18.0166, 2018/05/24 (n=1)
Boshoff et al., 2020, Infected Leaf, 2 Leaf, Yellow Rust, 18.0167, 2018/05/25 (n=1)
Boshoff et al., 2020, Infected Leaf, 1 Leaf, Yellow Rust, 18.0169, 2018/11/04 (n=1)
Boshoff et al., 2020, Infected Leaf, 1 Leaf, Yellow Rust, 18.0171, 2018/11/04 (n=1)
Boshoff et al., 2020, Infected Leaf, 1 Leaf, Yellow Rust, 18.0172, 2018/11/04 (n=1)
Boshoff et al., 2020, Infected Leaf, 1 Leaf, Yellow Rust, 18.0173, 2018/11/04 (n=1)
Boshoff et al., 2020, Infected Leaf, 1 Leaf, Yellow Rust, 18.0175, 2018/11/04 (n=1)
Boshoff et al., 2020, Infected Leaf, 1 Leaf, Yellow Rust, 18.0177, 2018/11/04 (n=1)
Boshoff et al., 2020, Infected Leaf, 1 Leaf, Yellow Rust, 18.0178, 2018/11/04 (n=1)
Boshoff et al., 2020, Infected Leaf, 1 Leaf, Yellow Rust, 18.0183, 2018/11/04 (n=1)
Boshoff et al., 2020, Infected Leaf, 1 Leaf, Yellow Rust, 18.0184, 2018/11/04 (n=1)
0
500
1,000
1,500
B
study
High level tissue
Tissue
High level age
Age
High level stress-disease
Stress-disease
High level variety
Host Species
Host Variety
Sample IDs
Date Isolated
location
country
Infection Percentage
Fungicide Treatment
Collector
PST130_13650
Haustoria, Seedling, Yellow Rust, 08/21: Haustoria, 2008 (n=2)
Germinating Spores, N/A, Yellow Rust, 87/66: Germinating Spores, 1987 (n=3)
Haustoria, N/A, Yellow Rust, Pst 104E137A-: Haustoria, 1979 (n=3)
Germinating Spores, N/A, Yellow Rust, Pst 104E137A-: Germinated Spores, 1979 (n=6)
Ungerminated Spores, N/A, Yellow Rust, Pst 104E137A-: Ungerminated Spores, 1979 (n=3)
Haustoria, N/A, Yellow Rust, Pst 104E137A-: Haustoria Enriched, 1979 (n=3)
0
2,000
4,000
6,000
